# Unveiling the Regulatory Role of LncRNA MYU in Hypoxia-Induced Angiogenesis via the miR-23a-3p Axis in Endothelial Cells

**DOI:** 10.3390/cells13141198

**Published:** 2024-07-15

**Authors:** Xiankun Zhou, Mingxing Wen, Jinwei Zhang, Keren Long, Lu Lu, Long Jin, Jing Sun, Liangpeng Ge, Xuewei Li, Mingzhou Li, Jideng Ma

**Affiliations:** 1 State Key Laboratory of Swine and Poultry Breeding Industry, College of Animal Science and Technology, Sichuan Agricultural University, Chengdu 611130, China; scauzxk99@163.com (X.Z.); wenmx0126@163.com (M.W.); keren.long@sicau.edu.cn (K.L.); lu.lu@sicau.edu.cn (L.L.); longjin@sicau.edu.cn (L.J.); xuewei.li@sicau.edu.cn (X.L.); mingzhou.li@sicau.edu.cn (M.L.); 2 Chongqing Academy of Animal Sciences, Chongqing 402460, China; jinweizhang50@163.com (J.Z.); sunjing85026@163.com (J.S.); geliangpeng1982@163.com (L.G.); 3 Key Laboratory of Pig Industry Sciences, Ministry of Agriculture, Chongqing 402460, China; 4 Chongqing Key Laboratory of Pig Industry Sciences, Chongqing 402460, China

**Keywords:** HUVEC, MYU, miR-23a-3p, IL-8, angiogenesis

## Abstract

**Background:** Angiogenesis is essential for various physiological and pathological processes, such as embryonic development and cancer cell proliferation, migration, and invasion. Long noncoding RNAs (lncRNAs) play pivotal roles in normal homeostasis and disease processes by regulating gene expression through various mechanisms, including competing endogenous RNAs (ceRNAs) of target microRNAs (miRNAs). The lncRNA MYU is known to promote prostate cancer proliferation via the miR-184/c-Myc regulatory axis and to be upregulated in vascular endothelial cells under hypoxic conditions, which often occurs in solid tumors. In the present study, we investigated whether MYU might affect cancer growth by regulating angiogenesis in vascular endothelial cells under hypoxia. **Methods:** The expression of MYU-regulated miR-23a-3p and interleukin-8 (IL-8) in HUVEC cell lines was examined using qRT-PCR. The CCK-8 assay, EdU assay, wound-healing assay, and tube-formation assay were used to assess the effects of MYU on cell proliferation, migration, and tube formation of HUVEC cells in vitro. The dual-luciferase reporter assay was performed to examine the effects of miR-23a-3p on MYU and IL-8 expression. **Results:** We found that the overexpression of MYU and knockdown of miR-23a-3p in human umbilical vein endothelial cells (HUVECs) under hypoxia promoted cell proliferation, migration, and tube formation. Mechanistically, MYU was shown to bind competitively to miR-23a-3p, thereby preventing miR-23a-3p binding to the 3′ untranslated region of IL-8 mRNA. In turn, increased production of pro-angiogenic IL-8 promoted HUVEC proliferation, migration, and tube formation under hypoxia. **Conclusion:** This study identified a new role for lncRNA MYU as a ceRNA for miR-23a-3p and uncovered a novel MYU–miR-23a-3p–IL-8 regulatory axis for angiogenesis. MYU and/or miR-23a-3p may thus represent new targets for the treatment of hypoxia-related diseases by promoting angiogenesis.

## 1. Introduction

Angiogenesis is the process by which new capillaries are formed from pre-existing blood vessels through matrix remodeling and endothelial cell budding, proliferation, and migration [1,2,3]. As the primary conduits for oxygen and nutrient supply to tissues and the distribution of immune cells to sites of damage and infection, blood vessels play essential roles in the maintenance of normal tissue growth and physiology in vertebrates. Angiogenesis is thus involved in many physiological and pathological processes, including embryonic development [4], menstrual cycle [5], placental development [6], retinal health [7], wound healing [8], and tumor vascular growth [9]. Angiogenesis is regulated by a balance between inducing factors, which include vascular endothelial growth factor (VEGF) [10], angiopoietin [11], and FGF [12], and inhibiting factors, such as arresten [13], endostatin [14], angiostatin [15], and MMP [16]. Interactions between these factors are important for the regulation of angiogenesis, and an imbalance between them is responsible for the abnormal growth of microvessels in many diseases [17]. Although anti-angiogenic agents such as antibodies that target VEGF and its associated pathways are currently used for various clinical applications, they have shown limited benefit in many conditions, and there is a continuing need to develop additional drugs that can promote or inhibit angiogenesis.

Long noncoding RNAs (lncRNAs), usually defined as RNAs > 200 nucleotides in length that lack protein-coding potential, are increasingly recognized for their important roles in many biological processes. To date, the functions of a small subset of the thousands of lncRNAs in the human genome have been characterized, and multiple mechanisms of action have been identified. Firstly, lncRNAs act as signal molecules and are able to mark the space, time, developmental stage, and expression of gene regulation. Secondly, lncRNAs can act as decoy molecules to recruit other RNA-binding proteins and to regulate RNAs away from their functional sites. A further example of decoys is the lncRNA decoy for miRNA target sites. Thirdly, lncRNAs can act as guide molecules, bind proteins or bind specific regions of DNA, and guide protein–RNA complexes to localize to the site to be determined to play a guiding role. Finally, lncRNAs can function as scaffolds for complexes that bind multiple substrates simultaneously [18]. Notably, it has been established that some lncRNAs can regulate angiogenesis under hypoxic conditions; these include H19 [19], MALAT1 [20], GATA6-AS [21], and MEG3 [22,23], which are regulated by hypoxia in vascular endothelial cells. Recently, a new mechanism of action for lncRNAs has been described; namely, as competitive endogenous RNAs (ceRNAs). ceRNAs promote mRNA stability and/or translation by binding to microRNA (miRNAs), thereby acting as molecular sponges that block the binding of miRNAs to their target mRNAs. For instance, in human umbilical vein endothelial cells (HUVECs) under hypoxia, lncRNA HIF1A-AS2 promotes the upregulation of the key hypoxia-related transcription factor hypoxia-inducible factor-1α (HIF-1α) by binding to miR-153-3p, thereby promoting angiogenesis [24]. Conversely, the loss of function of lncRNA UCA1 causes the upregulation of miR-195, which leads to the downregulation of components of the MEK/ERK and mTOR signaling pathways and CCND1 expression, thus inhibiting the growth of microvascular endothelial cells and vessel formation [25]. LncRNA NEAT1 promotes endothelial cell survival and angiogenesis by binding to miR-377 and promoting the expression of SIRT1, VEGF-A, and BCL-XL [26]. LINC01693 acts as a ceRNA of miR-302d and promotes angiogenesis in HUVECs by counteracting the miR-302d-mediated inhibition of CXCL12 [27]. In glioma vascular endothelial cells, overexpression of the lncRNA PVT1 enhances the expression of the autophagy proteins ATG7 and Beclin1 by targeting miR-186, and the subsequent induction of protective autophagy promotes tumor cell proliferation, migration, and angiogenesis [28]. MEG3 also plays an important role in the proliferation and angiogenesis of vascular endothelial cells by acting as a ceRNA and negatively regulating miR-9 [29].

A recent study showed that the lncRNA MYU is upregulated by the Wnt/c-Myc signaling pathway and promotes cell cycle progression by interacting with the RNA-binding protein hnRNP-K and stabilizing CDK6 expression [30]. Interestingly, MYU acts as a tumor promoter in colorectal cancer (CRC) but as a tumor suppressor in gastric cancer [31]. MYU, a product of the antisense strand of the *VPS9D1* gene, was among many functional lncRNAs shown to be associated with HUVEC angiogenesis under hypoxic conditions [32]. In that study, Moreau et al. investigated the lncRNA profiles of hypoxic human primary endothelial cells using Global Nuclear Run-On Sequencing (GRO-Seq), and they identified 1763 differentially expressed lncRNAs, of which 544 were upregulated, and 350 were downregulated (>2-fold) under hypoxia compared with normoxia. Most of these differentially expressed lncRNAs have yet to be functionally validated. In the present study, we selected MYU for further investigation of its mechanism of action using HUVECs, which respond to pro-angiogenic factors, such as VEGF, HIF-1α, and IL-8, and are a widely used model for studying the angiogenic process. We investigated the role of MYU in regulating HUVEC proliferation, migration, invasion, and angiogenesis under hypoxic conditions, and identified MYU as a ceRNA for miR-23b-3p, which, in turn, controls IL-8 expression.

## 2. Materials and Methods

### 2.1. TCGA Database Analysis

The Cancer Genome Atlas (TCGA, https://portal.gdc.cancer.gov/repository accessed on 23 June 2021) mainly includes clinical data, genomic variations, mRNA expression, miRNA expression, methylation, and other data on various human cancers, which is a very important data source for cancer researchers. First, we downloaded the HTSeq-FPKM data of 22 common cancers by TCGA. The expression of the MYU gene for each sample of the 22 common cancers was then extracted using the R language and subjected to statistical analysis.

### 2.2. In Vitro Translation Analysis

The constructed MYU overexpression plasmid was transfected into the HeLa cell line and cultured in a 5% CO_2_ incubator for 48 h. Total protein was extracted using Cell lysis buffer for Western and IP (Beyotime, Shanghai, China), and protein concentration was determined by Enhanced BCA Protein Assay Kit (Beyotime, Shanghai, China), followed by constant voltage electrophoresis at 120 V for 2 h using NUPAGE 10% BT GEL 1.0 MM 10 W 10 PER BOX (Invitrogen, Grand Island, NY, USA), then staining was conducted by using the Coomassie brilliant blue rapid staining solution (Solarbio, Beijing, China), and photographs were taken after the end of staining.

### 2.3. Cell Culture and Transfection

HUVECs were obtained from the Institute of Cardiovascular Diseases, University of South China (Hengyang, Hunan, China) and cultured in DMEM (Hyclone, Logan, UT, USA) containing 10% fetal bovine serum (FBS; GIBCO, Grand Island, NY, USA), penicillin, and streptomycin (×100). The cells were maintained at 37 °C in a 5% CO_2_ atmosphere, and the medium was changed every 2 to 3 days. Hypoxia was induced by placing cells in a 2.5 L anaerobic jar (MGC, Tokyo, Japan) with an AnaeroPack-Anaero (MGC, Tokyo, Japan), which generates hypoxic conditions (defined as <1% O_2_, 5% CO_2_, 94% N_2_) within 1 h. Normoxia was defined as 5% CO_2_ in the air.

Cells were transfected with a miR-23a-3p mimic, a control negative control sequence (NC), or a miRNA inhibitor (RIBOBIO, Guangzhou, Guangdong, China) using HiPerFect transfection reagent (Qiagen, Dusseldorf, North Rhine-Westphallen, Germany). Overexpression plasmids with or without the miRNA mimic were transfected into cells using Lipofectamine3000 (Invitrogen, Grand Island, NY, USA).

### 2.4. Plasmid Construction

The MYU overexpression plasmid was constructed by cloning the complete sequence of MYU into the pcDNA3.1 (+) vector via KpnI and EcoRI sites. For the luciferase reporter assays, MYU sequences containing either the wild-type putative binding site for miR-23a-3p (MYU-WT) or one in which the putative binding site was mutated (MYU-MUT) were cloned into the pmirGLO Dual-Luciferase miRNA Target Expression Vector (Promega, Madison, WI, USA). Additional pmirGLO plasmids were constructed, in which the luciferase expression was driven by the wild-type 3′ untranslated region (3′UTR) of IL-8 (IL-8-WT) or one in which the putative miR-23a-3p binding site was mutated (IL-8-MUT).

### 2.5. Quantitative Reverse-Transcription Polymerase Chain Reaction (qRT-PCR)

Total RNA was extracted from the cells using TRIzol reagent (TaKaRa, Tokyo, Japan) according to the manufacturer’s instructions. Samples of 1 μg of RNA were reverse-transcribed using a PrimeScript RT Reagent Kit with gDNA Eraser (Perfect Real Time; TaKaRa), and aliquots of cDNA were amplified by qPCR using TB Green Premix Ex Taq II (TliRNaseH Plus; TaKaRa) and a Bio-Rad CFX Connect™ quantitative fluorescence PCR detection system according to the manufacturer’s instructions. The reaction conditions were as follows: 95 °C for 30 s followed by 40 cycles of 95 °C for 5 s and 60 °C for 30 s. Glyceraldehyde 3-phosphate dehydrogenase (*GAPDH*) mRNA was amplified as an internal control. The primer sequences for qPCR are listed in Table 1.

### 2.6. Isolation of Nuclear and Cytoplasmic RNA

Nuclear and cytoplasmic RNA fractions were isolated from HUVEC cells and purified using a Nuclei EZ Prep kit (Sigma, San Francisco, CA, USA). Nuclei were stained using the trypan blue solution, and the purity of the final nuclei was determined by microscopy.

### 2.7. Dual-Luciferase Reporter Assay

Cells were plated in 48-well plates and transfected with the appropriate pmirGlo vector and control or miRNA mimic using Lipofectamine 3000 (Invitrogen) according to the manufacturer’s instructions. The luciferase activity was measured at ~36–48 h after transfection using a Dual-Luciferase Assay kit (Beyotime, Shanghai, China) according to the manufacturer’s manual.

### 2.8. CCK-8 Assay

To assess cell proliferation, we employed the CCK-8 assay, a colorimetric method that quantifies cell numbers by measuring the absorbance of a yellow-colored compound formed from formazan within the cells. HUVECs were seeded into 96-well plates and subjected to the indicated treatments (n = 5). At baseline (0 h) and after 6, 12, 24, and 48 h of incubation, 10 μL of CCK-8 reagent (Beyotime) was added to each well, and the incubation was continued for 2 h. The absorbance at 450 nm was then read in a microplate reader (BioTek, Burlington, VT, USA).

### 2.9. EdU Assay

The EdU assay, on the other hand, is a method for detecting cellular DNA synthesis, using fluorescent labeling of newly synthesized DNA to evaluate the proliferative activity of cells. HUVECs were seeded into 96-well plates, subjected to the indicated treatments, and stained using a Cell-Light EdU Apollo567 In Vitro Kit (RIBOBIO) according to the manufacturer’s manual. The cells were examined using an inverted fluorescence microscope (Leica, Wetzlar, Hesse, Germany). Images of whole cells and nuclei were captured, and the proportion of replicating cells was calculated.

### 2.10. Wound-Healing Assay

HUVECs were seeded into 12-well plates and grown to confluence. A wound was inflicted by passing a 10 μL pipette tip across the monolayer. The non-adherent cells were removed, and the cells were photographed, incubated at 37 °C for 24 h, and photographed again. Wound closure was calculated from the images of the photographs captured at 0 and 24 h. The experiments were repeated at least three times.

### 2.11. Tube-Formation Assay

Matrigel (Corning, Corning, NY, USA) was added to the 24-well plates and allowed to gel at 37 °C in a 5% CO_2_ atmosphere for 30–60 min. HUVECs (1.2 × 10^5^ cells/500 μL) were added to the wells, and the plates were incubated at 37 °C under hypoxic conditions. Tube formation was evaluated using an inverted microscope (Leica). Image-Pro Plus 5.0 (Media Cybernetics, Rockville, MD, USA) software was used to analyze the tube lengths and branches in 10 randomly selected visual fields.

### 2.12. Statistical Analysis

Data are expressed as the mean ± standard deviation (SD) of triplicates unless noted. Differences between the two groups were evaluated by a two-tailed Student’s *t*-test, and differences among multiple groups were evaluated by two-way ANOVA. A two-tailed *p*-value < 0.05 was considered significant. All analyses were performed using Prism 8.0 (GraphPad Software, San Diego, CA, USA).

## 3. Results

### 3.1. Expression Pattern of MYU and Upregulation under Hypoxia In Vitro

**Figure 1:**
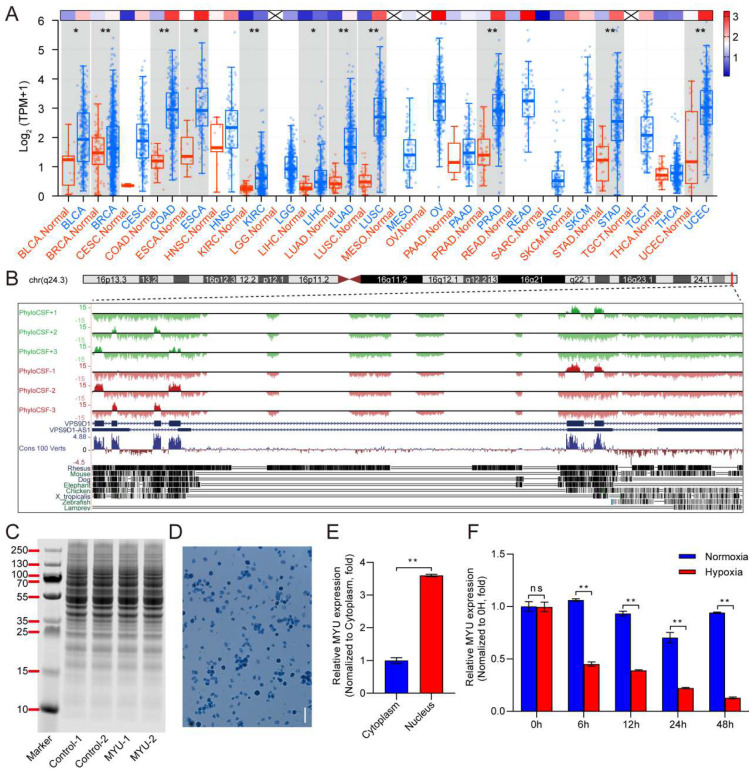
Expression pattern of MYU. (**A**) MYU expression levels in various tumor types and corresponding normal tissues in TCGA datasets. The corresponding heatmap represents the average expression of MYU genes in each type of cancer tissue. (**B**) Genomic landscape of MYU using the UCSC Genome Browser. The chromosomal location of MYU and examples of its conservation in vertebrates are shown. (**C**) In vitro translation experiments show no evidence of protein production by MYU. (**D**) Trypan blue staining of nuclei isolated from HUVECs. (**E**) qRT-PCR analysis of MYU in the nuclear and cytoplasmic fractions of HUVECs. (**F**) qRT-PCR analysis of MYU in HUVECs cultured under hypoxic or normoxic conditions at various times. All experiments except (**F**) were conducted under normoxic conditions. Data represent the mean ± SD (*n* = 3). * *p* < 0.05, ** *p* < 0.01, ns = not significant.

To determine whether MYU (VPS9D1-AS1) might be involved in angiogenesis during cancer, we examined its expression levels in different tumor tissues compared with the corresponding normal tissues by interrogating the RNA-seq datasets of 22 common malignancies from The Cancer Genome Atlas database. The expression of MYU was higher in tumor tissues than in normal tissues for all 22 malignancies, and the difference reached significance for bladder urothelial carcinoma (*p* = 0.0011), breast invasive carcinoma (*p* = 0.0008), colon adenocarcinoma (*p* < 0.0001), esophageal carcinoma (*p* = 0.0028), kidney renal clear cell carcinoma (*p* < 0.0001), liver hepatocellular carcinoma (*p* = 0.0039), lung adenocarcinoma (*p* < 0.0001), lung squamous cell carcinoma (*p* < 0.0001), prostate adenocarcinoma (*p* < 0.0001), stomach adenocarcinoma (*p* < 0.0001), and uterine corpus endometrial carcinoma (*p* = 0.0002) (Figure 1A). Because hypoxia-stimulated angiogenesis is a common feature of many solid tumors, we speculated that the upregulation in MYU may be associated with the induction of angiogenesis under hypoxic conditions.

Using the UCSC Genome Browser, we determined that the MYU sequence was highly conserved across species (Figure 1B). PhyloCSF analysis indicated the possibility that MYU may have potential protein-coding capacity (Figure 1B). However, the results of the in vitro translation experiments showed that no additional protein bands were identified in the HeLa cell extracts in the presence of MYU compared with the controls (Figure 1C), indicating that MYU transcripts are unlikely to be protein-coding. To determine the subcellular localization of MYU, we isolated the nuclei (Figure 1D) and cytoplasmic fractions from HUVECs and performed a qRT-PCR analysis of MYU. This analysis revealed that most of the MYU transcripts were localized in the nucleus (Figure 1E). We next investigated whether the MYU expression was altered in HUVECs under hypoxic conditions (<0.1% O_2_), which might support a potential role for this lncRNA in promoting tumor angiogenesis. Notably, while the MYU expression was progressively downregulated in cells cultured in hypoxia conditions, the MYU levels were essentially maintained for up to 48 h under normoxic (Figure 1F). This finding is consistent with the results of Moreau et al. [32], showing the downregulation of MYU in HUVECs in response to hypoxia.

### 3.2. HUVEC Proliferation, Migration, and Angiogenesis under Hypoxia Are Promoted by MYU

**Figure 2:**
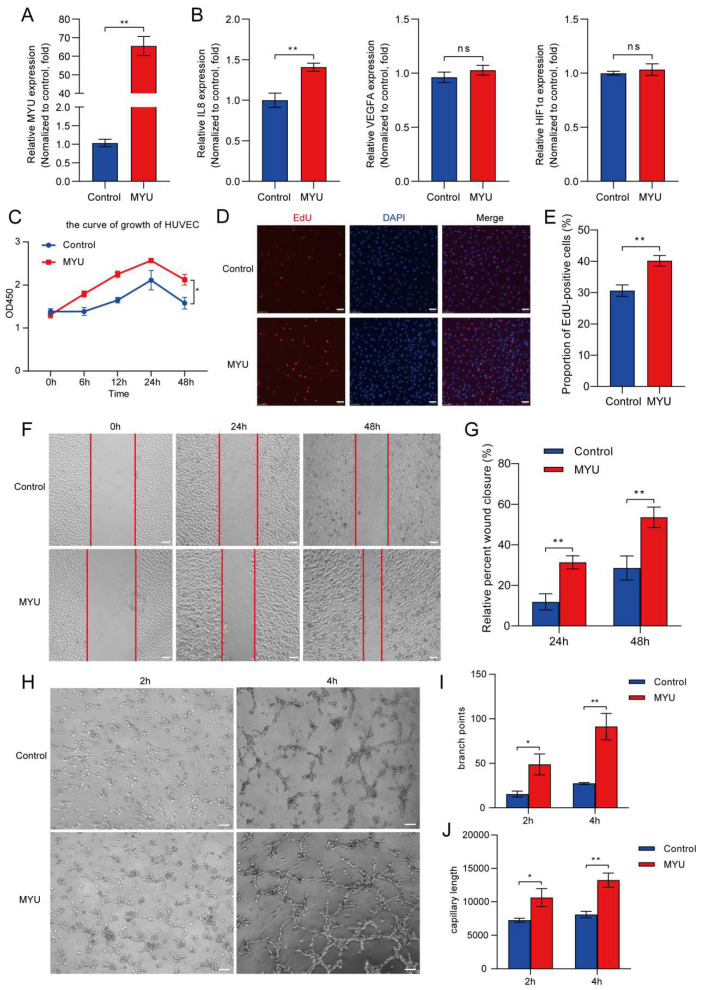
MYU is required for HUVEC proliferation, migration, and angiogenesis. (**A**,**B**) qRT-PCR analysis of MYU (**A**), IL-8, VEGFA, and HIF-1α (**B**) in HUVECs transfected with an empty vector or an MYU expression vector. (**C**–**E**) Cell proliferation of control and MYU-overexpressing HUVECs were detected with CCK-8 (**C**) and EdU (**D**,**E**) assays. Scale bar: 10.0 μm. (**F**,**G**) Images (**F**) and quantification (**G**) of migration of control or MYU-overexpressing HUVECs. Wound-healing assays were performed in a serum-free medium. Scale bar: 10.0 μm. (**H**–**J**) Images (**H**) and quantification of branching (**I**) and tube length (**J**) by control or MYU-overexpressing HUVECs cultured in Matrigel-coated wells. Scale bar: 10.0 μm. All experiments were conducted under hypoxic conditions. Data represent the mean ± SD (*n* = 3). * *p* < 0.05, ** *p* < 0.01, ns = not significant.

To investigate the role of MYU in angiogenesis under hypoxic conditions, we transfected HUVECs with an MYU overexpression vector or empty vector (control) and confirmed that the expression of MYU was increased (~65-fold) by qRT-PCR (Figure 2A). Next, we analyzed the expressions of angiogenesis-related factors VEGF, HIF-1α, and IL-8 in the control and MYU-overexpressing HUVECs under hypoxia. We observed that the expression of IL-8 mRNA, but not VEGF or HIF-1α mRNA, was significantly increased by MYU overexpression (Figure 2B, *p* < 0.01). An evaluation of cell proliferation under hypoxia using the CCK-8 and EdU proliferation assays indicated that MYU overexpression also increased HUVEC cell proliferation compared to the control cells (*p* < 0.05 and *p* < 0.01, respectively; Figure 2C–E). Similarly, the migration of HUVECs under hypoxia was promoted by MYU overexpression, as measured using the wound-healing assays (Figure 2F,G, *p* < 0.01). Finally, we performed tube-formation assays of HUVECs in Matrigel-coated wells and found that MYU overexpression significantly promoted not only tube formation but also branching under hypoxic conditions (Figure 2H–J, *p* < 0.01). These results indicated that MYU overexpression can promote HUVEC proliferation, migration, and angiogenesis during hypoxia.

### 3.3. MYU Overexpression Downregulates miR-23a-3p in HUVECs under Hypoxia

**Figure 3:**
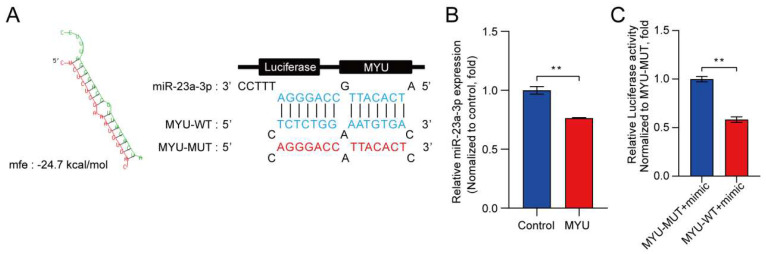
Interaction between MYU and miR-23a-3p in HUVECs. (**A**) Sequences of potential binding sites between MYU and miR-23a-3p, predicted by RNAhybrid (https://bibiserv.cebitec.uni-bielefeld.de/rnahybrid accessed on 28 July 2021) and luciferase reporter constructs, showing the location of the putative miR-23a-3p binding site in the wild-type (MYU-WT) and mutated (MYU-MUT) sequences in pmirGLO vector. (**B**) qRT-PCR analysis of miR-23a-3p levels in HUVECs transfected with a control or MYU overexpression vector. (**C**) Relative luciferase activity in HUVECs co-transfected with pmirGLO containing either the WT-MUT or MUT-MYU vectors and a miR-23a-3p mimic. Fig. B experiments were conducted under hypoxic conditions. Data represent the mean ± SD (*n* = 3). ** *p* < 0.01, ns = not significant.

Next, we probed the pro-angiogenic mechanism of action of MYU in HUVECs by RNAhybrid (https://bibiserv.cebitec.uni-bielefeld.de/rnahybrid accessed on 28 July 2021) for miRNAs with sequences complementary to MYU. We found that MYU contains a putative binding site for miR-23a-3p (Figure 3A). The qRT-PCR analysis of HUVECs indicated that miR-23a-3p expression was significantly reduced in MYU-overexpressing HUVECs compared to the control HUVECs under hypoxia (Figure 3B, *p* < 0.05), which supported a potential interaction between the two RNAs. To confirm this, we performed luciferase reporter assays in which HUVECs were transfected with an MYU mimic together with either a luciferase plasmid containing the WT-MYU sequence or MUT-MYU sequence in which the putative miR-23a-3p binding site was disrupted (Figure 3A). Notably, the luciferase activity was significantly reduced in cells overexpressing the miR-23a-3p mimic together with the MYU-WT plasmid compared with the cells expressing the MYU-MUT plasmid (*p* < 0.01, Figure 3C). These data demonstrated that MYU contains a functional miR-23a-3p binding site.

### 3.4. HUVEC Proliferation, Migration, and Angiogenesis under Hypoxia Is Inhibited by miR-23a-3p Overexpression

**Figure 4:**
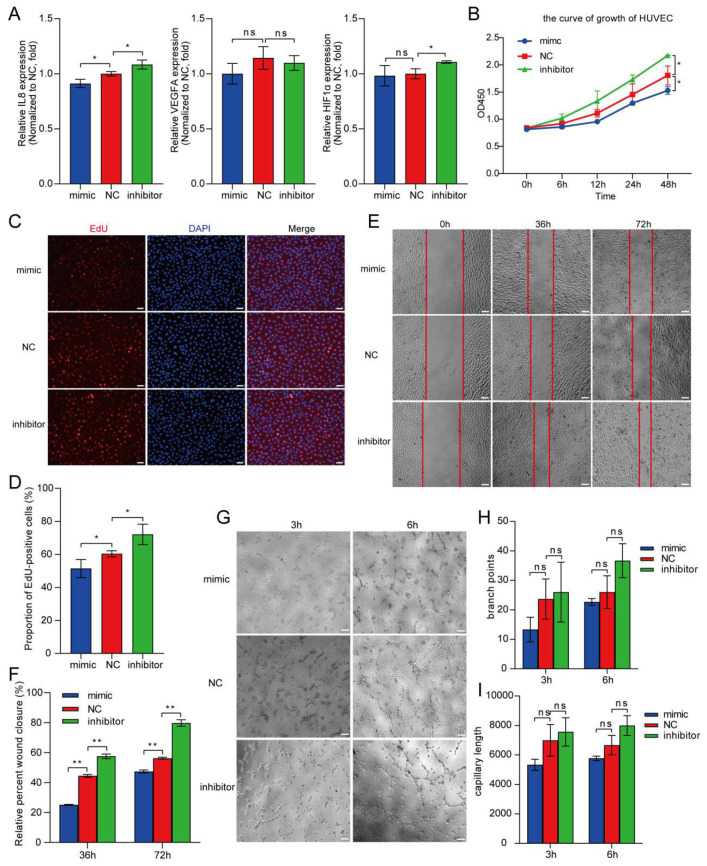
miR-23a-3p is required for HUVEC proliferation, migration, and angiogenesis under hypoxia. (**A**) qRT-PCR analyses of IL-8, VEGFA, and HIF-1α mRNA in HUVECs transfected with a control sequence (NC), a miR-23a-3p mimic, or a miR-23a-3p inhibitor. (**B**–**D**) HUVEC cell proliferation was detected using CCK-8 (**B**) and EdU (**C**,**D**) assays. Scale bar: 10.0 μm. (**E**,**F**) Migration of HUVECs in wound-healing assays performed in serum-free medium. Scale bar: 10.0 μm. (**G**–**I**) Tube formation and branching of HUVECs in Matrigel-coated wells. Scale bar: 10.0 μm. All experiments were conducted under hypoxic conditions. Data represent the mean ± SD (n = 3). * *p* < 0.05, ** *p* < 0.01, ns = not significant.

While miR-23a-3p has been reported to enhance the proliferative capacity of tumor cells under hypoxic conditions, its specific role in HUVECs has not been fully elucidated. Therefore, to investigate the role of miR-23a-3p in HUVEC angiogenesis, we transfected HUVECs with a miR-23a-3p mimic, a negative control sequence (NC), or a miR-23a-3p inhibitor, and incubated the cells under hypoxic conditions. The qRT-PCR analyses of VEGF, HIF-1α, and IL-8 revealed that the IL-8 mRNA levels were downregulated modestly but significantly by the miR-23a-3p mimic compared with the NC (*p* < 0.05) and were upregulated significantly by the miR-23a-3p inhibitor compared with the NC (Figure 4A, *p* < 0.01). Interestingly, the VEGFA mRNA levels were unaffected by the modulation of miR-23a-3p expression, whereas HIF-1α was upregulated by the miR-23a-3p inhibitor but unaffected by the mimic (Figure 4A). These results suggested that, like MYU, miR-23a-3p regulates the expression of angiogenic factors in HUVECs exposed to hypoxia. In addition, we found that the HUVEC cell proliferation measured with CCK-8 and EdU assays (Figure 4B–D), migration measured with wound-healing assays (Figure 4E,F), and angiogenesis measured with tube-formation assays (Figure 4G,H) were all inhibited by the miR-23a-3p mimic and promoted by the miR-23a-3p inhibitor compared with the control cells. Collectively, these results indicated that the upregulation and downregulation of miR-23a-3p inhibit and promote, respectively, HUVEC proliferation, migration, and angiogenesis under hypoxia.

### 3.5. Identification of an MYU–miR-23a-3p–IL-8 Axis That Regulates Angiogenesis under Hypoxia

**Figure 5:**
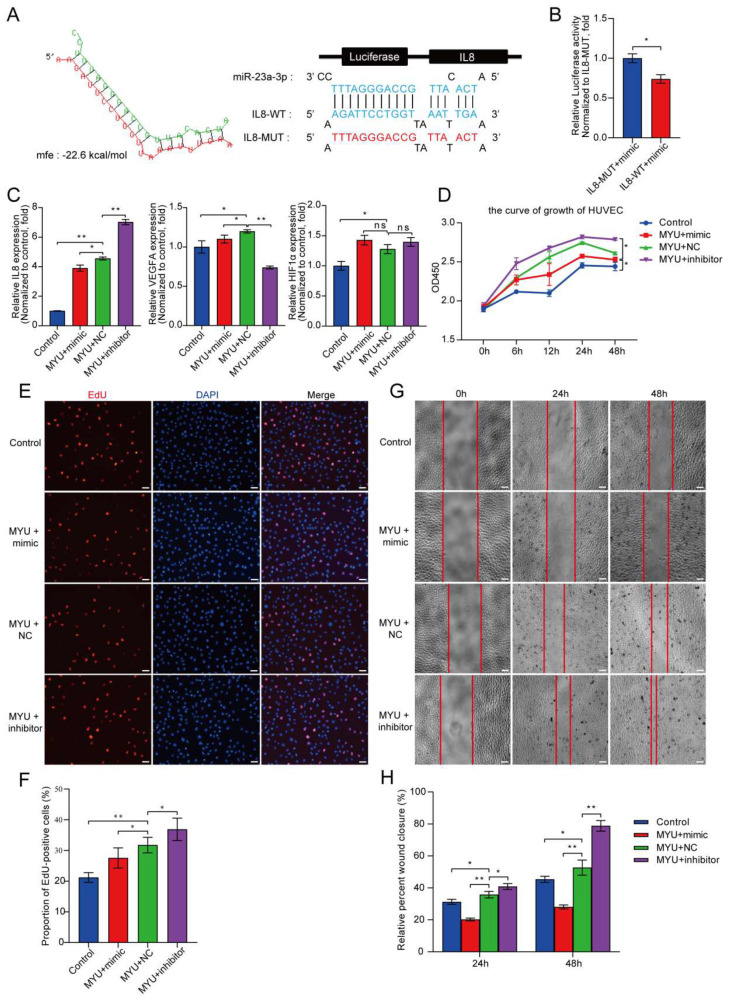

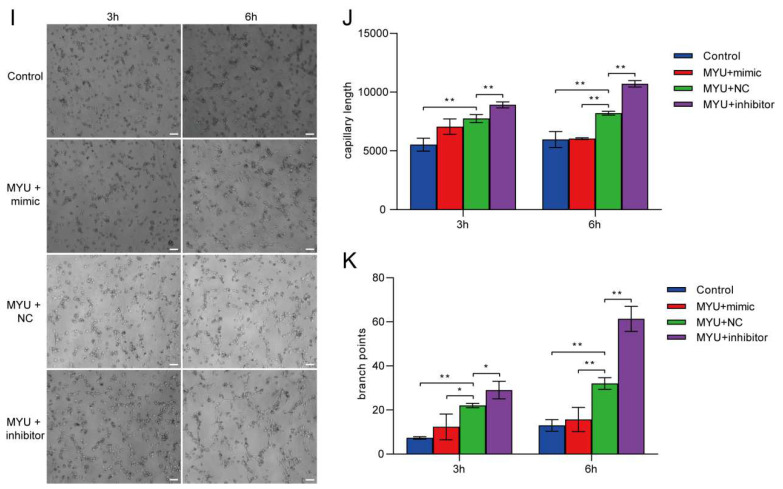
The MYU–miR-23a-3p–IL-8 axis regulates angiogenesis under hypoxia. (**A**) The potential binding site sequences between IL8 3′UTR and miR-23a-3p predicted by RNAhybrid (https://bibiserv.cebitec.uni-bielefeld.de/rnahybrid accessed on 28 July 2021) and luciferase reporter constructs showing the location of the putative miR-23a-3p binding site in the wild-type and mutated IL-8 3′UTR. (**B**) Relative luciferase activity in HUVECs co-transfected with pmirGLO vectors and miR-23a-3p mimic. (**C**) qRT-PCR analyses of IL-8, VEGFA, and HIF-1α mRNA levels in HUVECs co-transfected with an MYU overexpression vector and miR-23a-3p mimic, miR-23a-3p inhibitor, or negative control (NC) sequence. (**D**–**F**) HUVEC cell proliferation was detected using CCK-8 (**D**) and EdU (**E**,**F**) assays. Scale bar: 100 μm. (**G**,**H**) Migration of HUVECs in wound-healing assays performed in serum-free medium. Scale bar: 10.0 μm. (**I**–**K**) Tube formation and branching of HUVECs in Matrigel-coated wells. Scale bar: 10.0 μm. Figure (**C**–**K**) experiments were conducted under hypoxic conditions. Data represent the mean ± SD (n = 3). * *p* < 0.05, ** *p* < 0.01, ns = not significant.

Having demonstrated that miR-23a-3p expression is negatively regulated by MYU, that IL-8 is negatively regulated by miR-23a-3p, and that IL-8 is positively regulated by MYU in HUVECs under hypoxia, we speculated that MYU may regulate angiogenesis by acting as a ceRNA for miR-23a-3p, thereby alleviating the repression of IL-8. To investigate this, we first employed RNAhybrid (https://bibiserv.cebitec.uni-bielefeld.de/rnahybrid accessed on 28 July 2021) and determined that IL-8 mRNA contains a potential binding site for miR-23a-3p in its 3′UTR region (Figure 5A). We next confirmed that miR-23a-3p could directly bind to IL-8 by using reporter assays. HUVECs were transfected with a miR-23a-3p mimic together with a luciferase reporter vector, driven by either the IL-8-WT 3′UTR or an IL-8-MUT 3′UTR containing mutations in the putative miR-23a-3p binding site (Figure 5A). Notably, luciferase activity was significantly suppressed in cells co-expressing the miR-23a-3p mimic and the IL-8-WT reporter compared with the mimic plus IL-8-MUT reporter (*p* < 0.05, Figure 5B), demonstrating that miR-23a-3p directly binds to the 3′UTR of IL-8 mRNA.

Next, we examined the functional effects of concomitant modulation of the expression of both miR-23a-3p and MYU in HUVECs under hypoxia. HUVECs were co-transfected with an MYU overexpression plasmid plus miR-23a-3p mimic, NC, or miR-23a-3p inhibitor. Interestingly, the qRT-PCR analyses of VEGF, HIF-1α, and IL-8 (Figure 5C) showed that the IL-8 mRNA expression in MYU-overexpressing HUVECs was significantly reduced by co-transfection with the miR-23a-3p mimic (*p* < 0.05) and significantly increased by co-transfection with the miR-23a-3p inhibitor (*p* < 0.01). Interestingly, the VEGFA mRNA levels were significantly downregulated upon co-transfer of MYU with the miR-23a-3p inhibitors, in contrast to our expected results. Alternatively, the mRNA expression level of HIF-1α was not affected by the co-transfection of MYU with miR-23a-3p (Figure 5C). Moreover, we observed the same pattern of effects of co-modulation of MYU and miR-23a-3p levels in the HUVEC functional assays. Thus, compared with MYU -overexpressing, NC-transfected HUVECs, and MYU-overexpressing HUVECs transfected with the miR-23a-3p mimic or inhibitor, these exhibited elevated or suppressed, respectively, cell proliferation (*p* < 0.05, Figure 5D–F), cell migration (*p* < 0.05, Figure 5G,H), and tube formation and branching (*p* < 0.01, Figure 5I–K). Taken together, these results indicate that miR-23a-3p modulation counteracts the impact of MYU overexpression on HUVEC proliferation, migration, and angiogenesis under hypoxia.

## 4. Discussion

In this study, we investigated the biological functions of lncRNA MYU in HUVECs under hypoxic conditions. We focused on IL8, VEGF, and HIF1α due to their pivotal roles in angiogenesis and hypoxia responses. Although VEGF is expressed at low levels in endothelial cells under physiological conditions, it is crucial for the angiogenic response of these cells. HIF1α, a key regulator under hypoxic conditions, influences the expression of various genes, including VEGF. IL8, as a pro-inflammatory cytokine, has a potential regulatory effect in hypoxia-induced angiogenesis. Our study was prompted by the demonstration that MYU promotes cancer cell survival during hypoxia. Yang et al. [31] examined the lncRNA expression profile of human CRC (Affymetrix Human Exon 1.0ST arrays) and identified MYU (VPS9D1-AS1) as a potential prognostic biomarker. MYU was also shown to be upregulated in cancer tissues compared to adjacent normal tissues and was suggested to be a key regulator that controls CRC carcinogenesis and development. Wang et al. [33] found that MYU may promote the proliferation of prostate cancer cells by competitively binding to miR-184 and increasing the expression of c-Myc. Kawasaki et al. [30] demonstrated that MYU binds to hnRNP-K and stabilizes CDK6 expression, thereby promoting the G1–S phase transition and playing a key role in the proliferation and tumorigenicity of colon cancer cells.

LncRNAs can regulate gene expression through various mechanisms, including chromatin remodeling, direct transcriptional regulation, and post-transcriptional processing [34,35,36]. LncRNAs that act as ceRNAs also play a major role in post-transcriptional regulation of protein-coding genes [35,36,37,38]. In the present study, we showed that MYU is upregulated in HUVECs during hypoxia, resulting in the downregulation of miR-23a-3p. These data initially suggested an interaction between the two RNAs, and we then confirmed that MYU directly binds to miR-23a-3p to act as a molecular sponge or ceRNA.

Several studies have demonstrated a crucial role for miR-23a-3p in hypoxia-related processes in cancer cells. For example, the upregulation of miR-23a-3p is known to inhibit melanoma cell proliferation, migration, and invasion and to promote apoptosis [39,40]. Wang et al. [41] showed that miR-23a-3p functions as a tumor suppressor in osteosarcoma, and its inhibitory effect was mainly mediated through the downregulation of SATB1. Chen et al. [42] showed that miR-23a-3p may inhibit proliferation and invasion and promote apoptosis of oral squamous cell carcinoma cells by targeting FGF2. Wang et al. [43] revealed that miR-23a-3p inhibits endothelial progenitor cell activity in patients with coronary artery disease by targeting EGFR.

In addition, miR-23a-3p has an established role in angiogenesis. Kwok et al. [44] demonstrated that ginsenoside-Rg1 can induce angiogenesis and that miR-23a-3p negatively regulates the MET tyrosine kinase receptor, indicating that miR-23a-3p can inhibit angiogenesis. Our results in the present study are consistent with those of Wu et al. [45], who showed that miR-23a-3p inhibited angiogenesis in HUVECs and zebrafish. However, a number of studies have also reported that miR-23a-3p can promote angiogenesis [46,47,48,49]. Zheng et al. [47] demonstrated that extracellular vesicles secreted by lung cancer cells can promote angiogenesis by transferring miR-23a-3p to HUVECs, resulting in a reduced expression of the tumor suppressor PTEN and increased cell proliferation and migration. There are several potential explanations for these apparently conflicting results, including the use of different treatment conditions and cell types between studies. Further work will be necessary to clarify these observations. In our study, Figure 5C unexpectedly shows a downregulation of VEGF expression when MYU is overexpressed in conjunction with the miR-23a-3p inhibitor. This may indicate a complex regulatory network where miR-23a-3p indirectly affects the VEGF levels or a cellular homeostatic mechanism to balance the angiogenic signals. Further research is needed to clarify these interactions, but our findings underscore the importance of considering multi-layered regulations in angiogenesis control. Furthermore, the discrepancies in VEGF and HIF1α expression between Figure 2B and Figure 5C indicate that changes in the MYU expression levels may significantly influence the regulatory effects of miR-23a-3p. These differences might uncover more complex molecular mechanisms in the control of angiogenesis, where the function of miR-23a-3p could be affected by alterations in the cellular environment due to the overexpression of MYU. This necessitates further in-depth investigations in future studies to comprehensively understand the roles of these critical molecules in angiogenesis.

**Figure 6:**
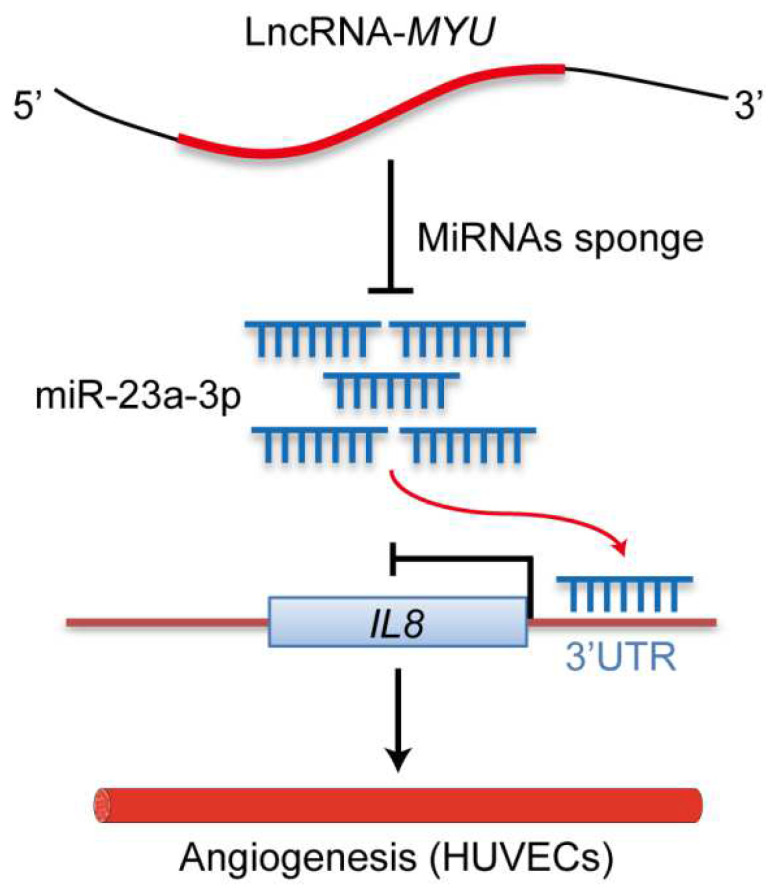
Model for the regulation of angiogenesis by the MYU–miR-23a-3p–IL-8 axis in HUVECs under hypoxia.

A model summarizing our results is shown in Figure 6. Our findings are consistent with a scenario in which MYU promotes the upregulation of IL-8 in HUVECs under hypoxia by acting as a molecular sponge for miR-23a-3p, thereby preventing miR-23a-3p-mediated repression of IL-8 translation and leading to enhanced angiogenesis, proliferation, and migration. The MYU–miR-23a-3p–IL-8 regulatory axis provides a novel potential therapeutic target and lays the foundation for the development of new strategies for the treatment of diseases and conditions that involve abnormal vascular growth.

## 5. Conclusions

Our study delves into the pivotal role of the long noncoding RNA MYU in hypoxia-induced angiogenesis, revealing its capacity to promote endothelial cell proliferation, migration, and tube formation through the miR-23a-3p/IL-8 signaling axis. The overexpression of MYU under hypoxic conditions robustly enhanced angiogenic activities by acting as a competing endogenous RNA (ceRNA) to miR-23a-3p, thereby alleviating its suppressive effect on IL-8 mRNA and promoting IL-8 expression. Furthermore, our findings suggest the potential of MYU and miR-23a-3p as therapeutic targets, offering a new theoretical foundation and direction for developing treatment strategies for hypoxia-related diseases. These discoveries not only deepen our understanding of the intricate regulatory mechanisms of angiogenesis but also provide valuable molecular targets for future clinical therapies.

## Figures and Tables

**Table 1:** Primer sequences for qRT-PCR.

Gene	Accession No.	Primers	Sequence
*MYU*	ENST00000562866.1	F	5′ ATGGGTAACCAGGGGTCAAG 3′
		R	5′ AGTAACAGTGGTAGAGCCGAC 3′
*IL-8*	ENST00000307407.8	F	5′ ACTGAGAGTGATTGAGAGTGGAC 3′
		R	5′ AACCCTCTGCACCCAGTTTTC 3′
*HIF-1α*	ENST00000539097.2	F	5′ TCCAAGAAGCCCTAACGTGT 3′
		R	5′ TGATCGTCTGGCTGCTGTAA 3′
*VEGF-A*	ENST00000372067.7	F	5′ ATGCGGATCAAACCTCACCA 3′
		R	5′ CACCAACGTACACGCTCCAG 3′
*GAPDH*	ENST00000396861.5	F	5′ GGAGCGAGATCCCTCCAAAAT 3′
		R	5′ GGCTGTTGTCATACTTCTCATGGA 3′
*U6*	NR_004394.1	F	5′ CTCGCTTCGGCAGCACA 3′
		R	5′ AACGCTTCACGAATTTGCGT 3′
*hsa-miR-23a-3p*	MIMAT0000078	F	5′ ATCACATTGCCAGGGATTTCC 3′

## Data Availability

The data presented in this study are available upon request from the corresponding author.

## Abbreviations

BLCA	Bladder urothelial carcinoma
BRCA	Breast invasive carcinoma
CESC	Cervical squamous cell carcinoma and endocervical adenocarcinoma
COAD	Colon adenocarcinoma
ESCA	Esophageal carcinoma
HNSC	Head and neck squamous cell carcinoma
KIRC	Kidney renal clear cell carcinoma
LGG	Brain lower grade glioma
LIHC	Liver hepatocellular carcinoma
LUAD	Lung adenocarcinoma
LUSC	Lung squamous cell carcinoma
MESO	Mesothelioma
OV	Ovarian serous cystadenocarcinoma
PAAD	Pancreatic adenocarcinoma
PRAD	Prostate adenocarcinoma
READ	Rectum adenocarcinoma
SARC	Sarcoma
SKCM	Skin cutaneous melanoma
STAD	Stomach adenocarcinoma
TGCT	Testicular germ cell tumors
THCA	Thyroid carcinoma
UCEC	Uterine corpus endometrial carcinoma.
